# The erector spinae plane block causes only cutaneous sensory loss on ipsilateral posterior thorax: a prospective observational volunteer study

**DOI:** 10.1186/s12871-020-01002-0

**Published:** 2020-04-20

**Authors:** Jingxiong Zhang, Yuting He, Shi Wang, Zhengjie Chen, Yu Zhang, Yuan Gao, Quanguang Wang, Yun Xia, Thomas J. Papadimos, Riyong Zhou

**Affiliations:** 1 Department of Anesthesiology, the First Affiliated Hospital of Wenzhou Medical University, Zhejiang, China; 2 Department of Anesthesiology, Ohio State University Wexner Medical Center, Columbus, OH USA

**Keywords:** Ultrasound, Nerve block, Erector Spinae plane, Cutaneous sensory block area, Volunteer study

## Abstract

**Background:**

Ultrasound-guided erector spine plane (ESP) block is widely used in perioperative analgesia for back, chest and abdominal surgery. The extent and distribution of this block remain controversial. This study was performed to assess the analgesia range of an ultrasound-guided ESP block.

**Methods:**

This prospective observational volunteer study consisted of 12 healthy volunteers. All volunteers received an erector spinae plane block at the left T5 transverse process using real-time ultrasound guidance. Measured the cutaneous sensory loss area (CSLA) and cutaneous sensory declination area (CSDA) using cold stimulation at different time points after blockade until its disappearance. The CSLA and CSDA were mapped and then calculated. The block range was described by spinous process level and lateral extension. The effective block duration for each volunteer was determined and recorded.

**Results:**

The cold sensory loss concentrates at T6-T9. The decline concentrates primarily at T4-T11. The lateral diffusion of block to the left side did not cross the posterior axillary line, and reached the posterior median line on the right. The area of cutaneous sensory loss was (172 ± 57) cm^2^, and the area of cutaneous sensory decline was (414 ± 143) cm^2^. The duration of cutaneous sensory decline was (586 ± 28) minutes.

**Conclusion:**

Ultrasound-guided erector spine plane block with 20 mL of 0. 5% ropivacaine provided a widespread cutaneous sensory block in the posterior thorax, but did not reach the anterior chest, lateral chest, or abdominal walls. The range of the blockade suggested that the dorsal branch of spinal nerve was blocked.

**Trial registration:**

Chinese Clinical Trial Registry, CHiCTR1800014438. Registered 13 January 2018

## Backgroud

Forero et al. [1] first reported the use of an ultrasound-guided ESP block, an interfascial plane block that successfully treats severe thoracic neuropathic pain. In ESP blockade, local anesthetic drugs are injected into the superficial or deep portion of the erector spinae using ultrasound guidance, then through the costotransverse foramina into the region of the spinal nerves and the origins of the dorsal and ventral rami [1]. It is simple, safe, and suitable to perform with an indwelling catheter to prolong postoperative analgesia, it may also prove to have the potential to replace the paravertebral block (PVB) in many clinical situations [2, 3].

Increasing cases of ultrasound-guided erector spinae plane block appeared in clinical applications [4, 5]. At the same time, in order to demonstrate the effect of ultrasound-guided ESP block, many clinical randomized controlled trials (RCT) have been conducted [3, 6–11]. Tulgar et al. [6] confirmed that bilateral ultrasound guided ESP block leads to effective analgesia and reduce analgesia requirement in first 12 h in patients undergoing laparoscopic cholecystectomy (LC). However, Chen et al. [7] demonstrated that ultrasound-guided multiple-injection PVB provided superior analgesia to intercostal nerve blocks (ICNB) and single-injection ESP block, while ICNB and single-injection ESP block were equally effective in reducing pain after thoracoscopic surgery. It has been reported that single-injection ESP block require more morphine [7]. Recently, a cadaveric study conducted by Aponte et al. [12] indicated that the dye and contrast agents diffuse in a cephalad-to-caudal direction in the dorsal region of T1-T11, and laterally diffuse to the ribs, as demonstrated by CT scan and anatomical observation. Moreover, no dye or contrast agent was observed to spread to the paravertebral region. Based on the contradiction between clinical trials and cadaveric research reports, it was of importance to perform a volunteer study to determine the range of the ESP block, and to clarify the applicability of the technique.

In this study, ultrasound-guided ESP blocks were performed in healthy volunteers, and the area of blockade was determined by dermal stimulation of cold sensors using ice. The main outcome measured was the range of sensory loss and decline in cutaneous cold sensation, and the secondary outcome measurement was the area of blockade and the duration of decreased cold sensation.

## Methods

### Volunteers

This observational volunteer study was approved by the University’s Institutional Review Board (IRB No.2017–22) and written informed consent was obtained from all subjects participating in the trial. The trial was registered prior to patient enrollment at Chinese Clinical Trial Registry (registration number: CHiCTR1800014438), Principal investigator: Jingxiong Zhang, Date of registration: December 11th, 2017. This study adheres to the applicable CONSORT guidelines.

All 12 volunteers that were enrolled provided written informed consent. The age of volunteers ranged from 25 to 50 years old, and the study was not gender limited. Volunteers required an American society of anesthesiologists (ASA) physical classification system status of I-II, a Body mass index (BMI) of 18.5–28.0 kg/m^2^, the area of study for cutaneous sensation needed to be intact in order to be confirmed by the cold stimulation test. Exclusion criteria were as follows: allergy to Ropivacaine or other drugs used in this trial, and inability to communicate, ASA grade ≥ 3, BMI less than 18.5 kg/m^2^ or greater than 28 kg/m^2^, abnormal platelet function or other coagulopathy, infectious disease of the skin sensation, abnormal skin sensation, spinal deformity, neuropathy, spinal cord disease, torso scar in area studied, and/or a history of abdominal surgery.

### Procedures

Intravenous access was established after the subject was admitted into the designated block ward, and routine monitoring was performed in all participants (noninvasive blood pressure, continuous electrocardiogram, and pulse oximetry). All participants were sedated with 1 to 2 mL of an intravenous mixture of 20 μg/mL fentanyl and 1 mg/mL midazolam.

**Fig. 1:**
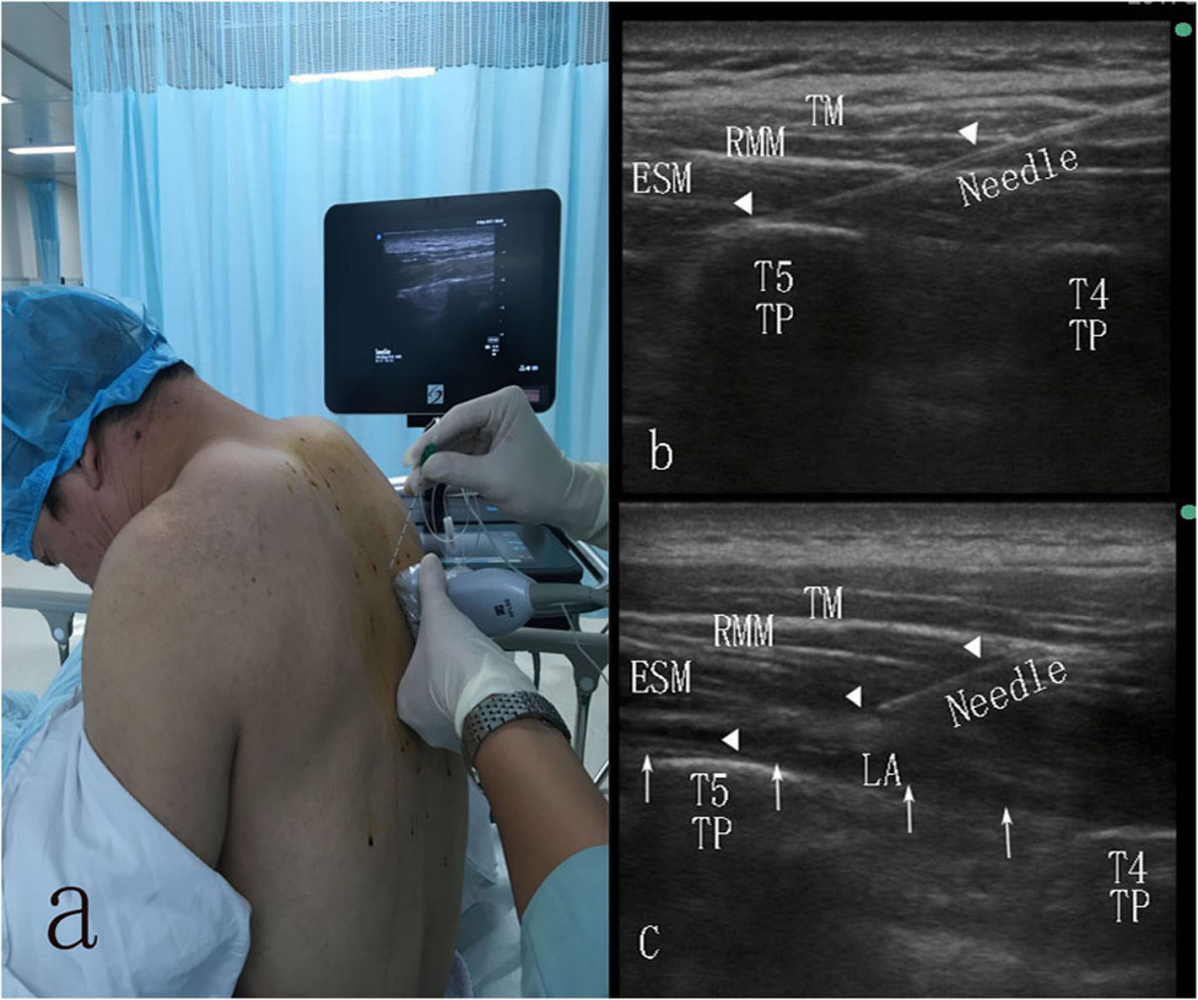
**a**: Positioning and scanning during erector spinae plane (ESP) block. The needle is inserted in a cephalad-to-caudal direction; **b**: The needle (triangle indicates) is inserted through the trapezius muscle (TM), rhomboid major muscle (RMM) and Erector Spinae Muscle (ESM), to the transverse process; **c**: An injection at this point creates a linear pattern of local anesthetic spread (arrows) that displaces the ESM downward

The volunteer was placed in a sitting position (Fig. 1a). The operator prepared a 80 mm 22G needle (Stimuplex® D; B. Braun Melsungen AG, Melsungen, Germany) and used a 38 mm wide high frequency 6-15MHZ ultrasound probe for high frequency linear sensors (Sonosite X-Porte; Sonosite Inc., Bothell, WA, USA), for block placement.

The apex of the left T5 transverse process (TP) was determined by using a high-frequency ultrasonic probe to identify the fifth rib, and then moved slowly in a medial direction to locate the transverse process. The apex of the T5 TP was identified by using the probe to determine the horizontal and sagittal sections of the T5 TP. The probe was placed in the longitudinal sagittal aspect to find the ultrasound image of the apex of T5 TP, approximately 3 cm lateral to the posterior medial line (Fig. 1b), and then the site was marked.

For the ESP blocks the operative area was disinfected, and the ultrasonic probe was covered with a sterile protective cover, and placed longitudinally at the marked point. The probe was adjusted to identify the three important layers of muscles (from posterior to anterior), i.e., trapezius, rhomboid, and erector spinae, and the optimal point for injection was identified (the apex of the left T5 TP). Local anesthesia with 1% lidocaine was instilled at the site of injection, 1 cm from the probe on the cephalad side. An 80 mm 22 G needle was inserted under ultrasound guidance (in plane), aiming towards the TP. Saline 1-2 ml was injected to determine the gap, and then 20 mL of 0. 5% ropivacaine (Naropin, AstraZeneca AB, Sweden) was injected. Another experienced anesthesiologist and the operator rechecked the needle tip and local anesthetic diffusion in the plane between the erector spinae and the TP with ultrasound (Fig. 1c).

### Measurement and calculation

**Fig. 2:**
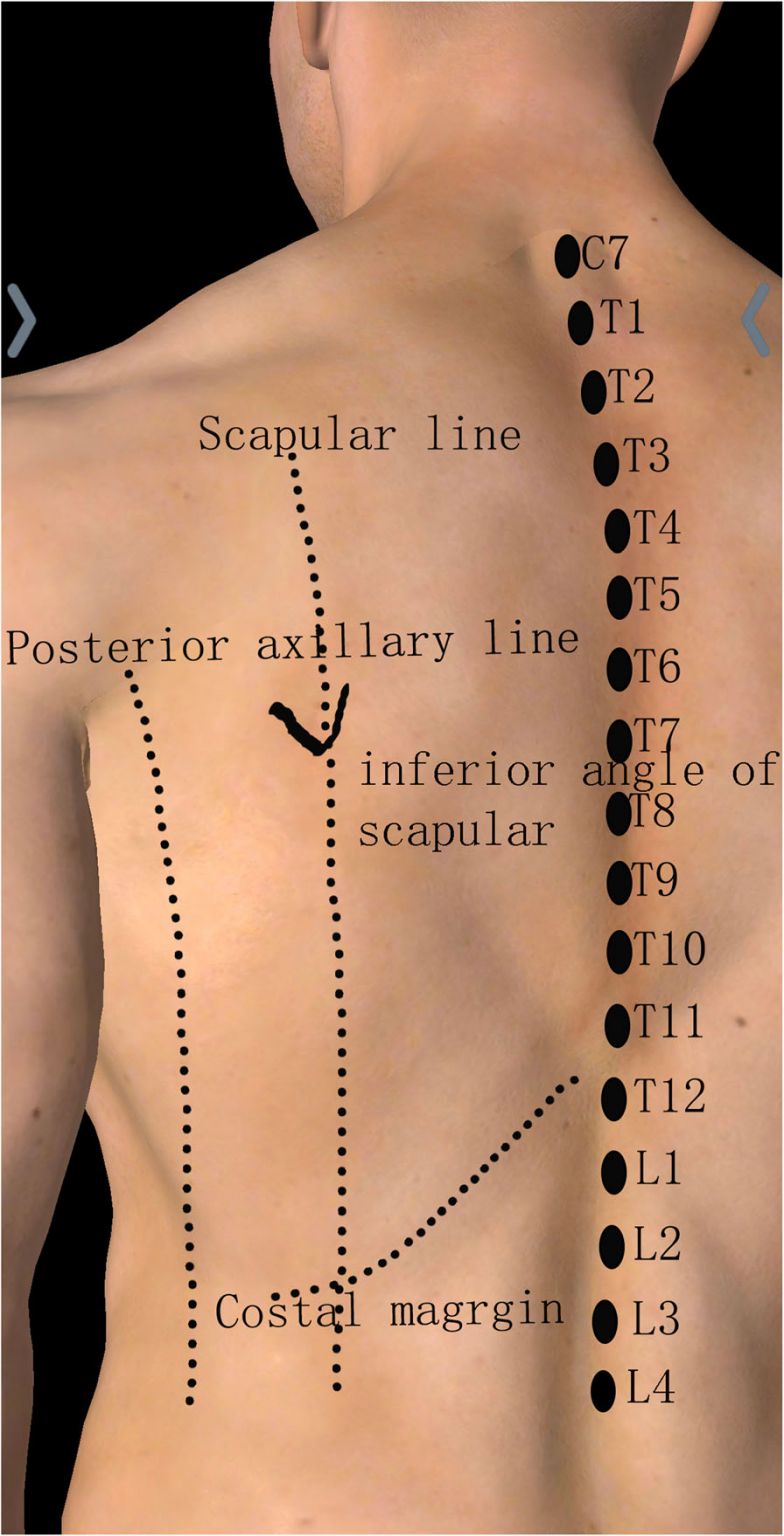
Marked lines and points

Important landmarks included: the posterior median line, the left scapular line, the left posterior line, the left 12th costal margin line, the left inferior angle of scapular, and the T1 to L4 spines (Fig. 2). For range of distribution measurements, the volunteers were kept in sitting position, and for the remaining time were in the supine position. The patients allowed to leave to urinate and to imbibe water as needed.

**Fig. 3:**
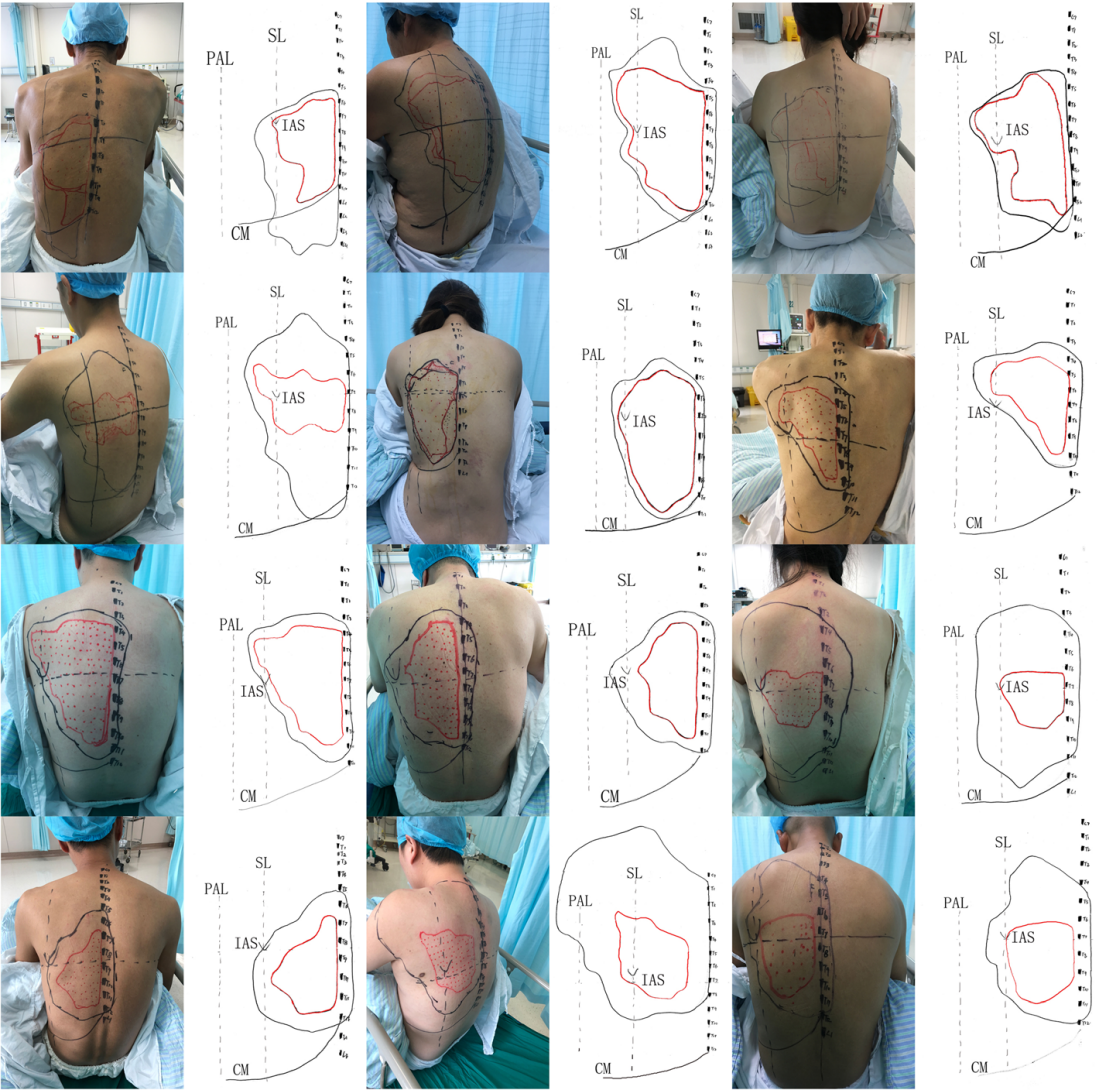
Photos and scanned transparencies; scapular line (SL), posterior axillary line (PAL); costal margin (CM); inferior angle of scapula (IAS)

The determination of the cutaneous sensory block area was done using cold stimulation (ice cubes) by an attending physician (other than the operator) at 0.5 h, 1 h, and every hour after injection. According to previous experiments, the block lasts for 10 h. Thus, 9 h after the block, we measured every 15 min until cutaneous sensory returns to normal. The time from the end of injection to the last measurement was considered the blockade duration, and was recorded as such. Sensation was evaluated on a 3-point scale: 0 = loss of cold sensation, 1 = decreased cold sensation compared with the sensation at unaffected areas, 2 = normal sensation. The cold stimulus was moved laterally at approximately 2 cm/s from the opposite posterior axillary line at 1 cm intervals and measurements ceased at the left midaxillary line. During application of the cold stimulus, areas of sensory change were marked on the skin, and the marked dots were connected in order to map the blocked area. We marked block levels 1 and 0 with black and red dots, respectively. During the observation period, when the block range was no longer expanding, we connected the black and red dots to map the CSDA and CSLA. Then photos were taken (and saved) (Fig. 3). We covered the CSDA and CSLA with a 37 cm*52 cm transparent rectangular film, and then transferred all surface landmarks/lines to it.

Afterwards, the transferred transparency was converted into a transparent digital-based images with a fixed pixel value (1057*1485) (size: 37 cm*52 cm) using a scanner (Fujitsu FI-7460), and then saved as a JPG format (Fig. 3). After completing the trial, we used the magnetic lasso tool to select the target area that needed to be calculated, and used the histogram toolbar to see/determine the pixel size of the selected area. The actual size of this digital picture was 1924 cm^2^ [target area pixel / total pixel (fixed pixel)]. We were able to calculate the CSLA and CSDA. The highest and lowest level of spinous processes were observed and recorded.

Any systemic toxicity events regarding local anesthetics, nausea, and vomiting during the block were recorded.

### Statistics analysis

Statistical analysis was performed with SPSS statistical software (version 21.0; SPSS, Chicago, Illinois) using the one-sample Kolmogorov-Smirnov normality test. The demographic data, cutaneous cold sensory area, and cutaneous sensory block duration, were expressed as mean ± SD.

## Results

### Demographic data

This study involved 12 volunteers, 8 were male. The ages ranged from 27 to 48 years, weight ranged from 62 kg to 79 kg, and height ranged from 158 cm to 177 cm. More details are in Table 1.

**Table 1:** Demographic data

Volunteer	Sex	Age (y)	Weight (kg)	Height (cm)	Body mass index (kg/m^2^)
#1	M	48	67	169	23.46
#2	F	47	62	158	24.84
#3	F	42	66	160	25.78
#4	M	27	73	180	22.53
#5	F	34	64	161	24.69
#6	M	38	60	169	21.01
#7	M	32	74	175	24.16
#8	M	30	73	176	23.57
#9	F	39	68	163	25.59
#10	M	31	72	173	24.06
#11	M	33	76	177	24.26
#12	M	35	79	175	25.80
Total	M:F(8:4)	36 ± 7	70 ± 6	170 ± 8	24.1 ± 1.4

### Block range of cold cutaneous sensation

The range of sensory loss was maximal at 1 h, with no further expansion. The cold sensory loss was from the T3 spinous process down to the T12 spinous process, and was most concentrated at T6-T9. Except for two volunteers, the lateral diffusion to the left side reached scapular line, did not cross the posterior axillary line; and to the right it reached 1 cm to the left of the posterior median line.

**Fig. 4:**
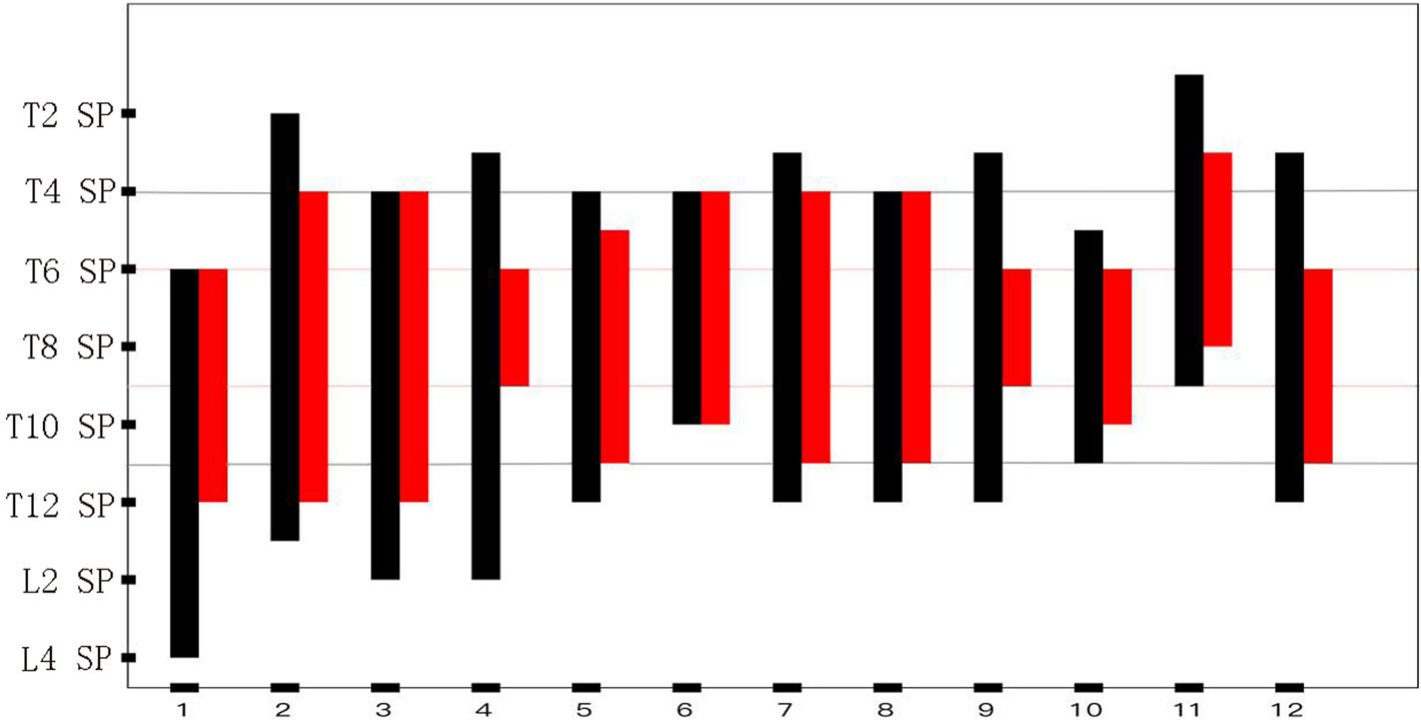
The cold sensory block reached the spinous process (SP). The black bars represent the range of cold sensation declination, the red bars represent the range of cold sensation loss

The cold sensory decline was from the T1 spinous process down to the L4 spinous process, and was concentrated at T4-T11. The lateral diffusion to the left side reached scapular line, did not cross the posterior axillary line, and to the right it reached the left of the posterior median line (Figs. 3 and 4).

### Block area

The average cutaneous sensory block area in the left posterior thorax was (172 ± 57) cm ^2^, and the decline area was (414 ± 143) cm ^2^ at 1 h.

### The duration of block

**Fig. 5:**
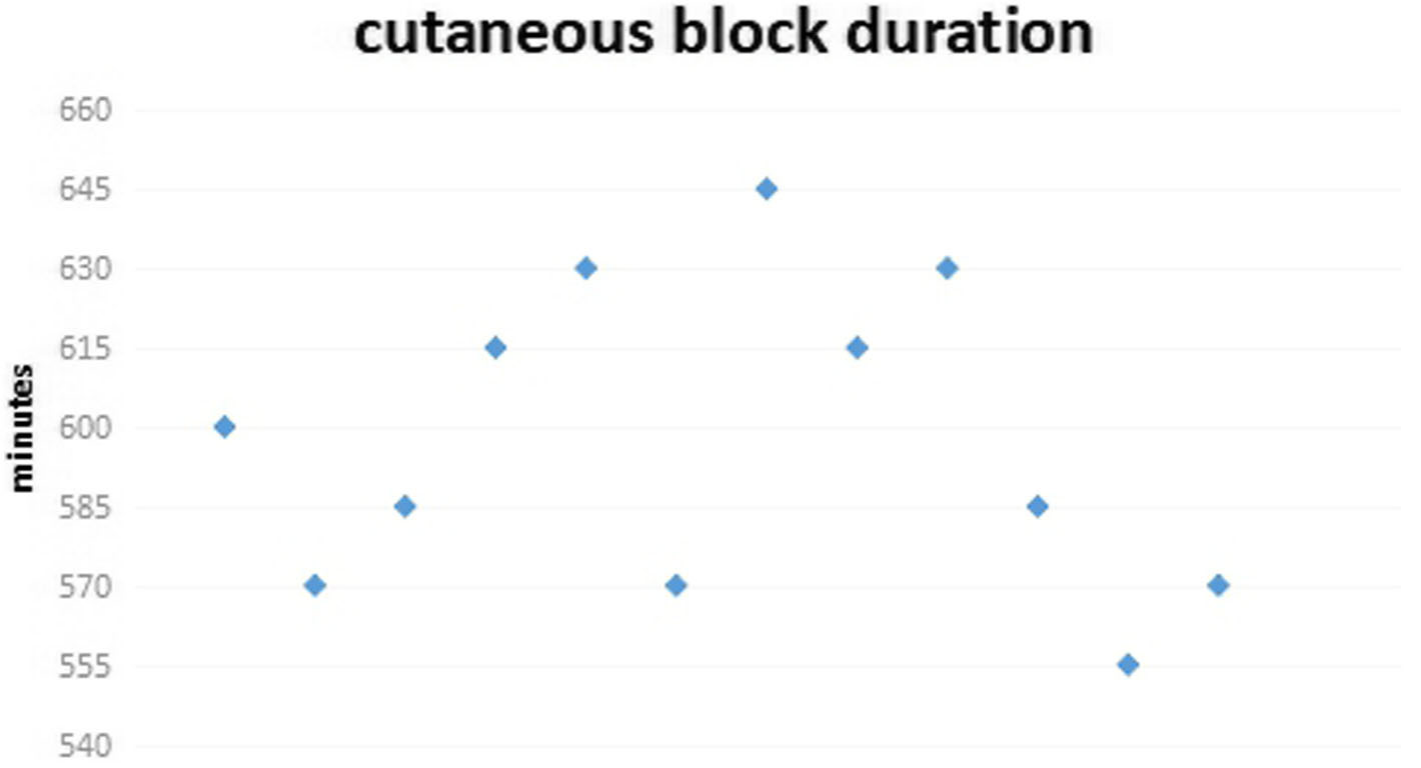
The duration of cold sensory block

The ESP block retarded the cold cutaneous sensation in posterior thorax for 555--645 min, and the duration of sensory decline was (586 ± 28) minutes (Fig. 5).

### Side effects and complications

During the operative/block process and subsequent measurement process, the general condition of the patients was very good, and there were no systemic toxicity events regarding local anesthetics, and there was no nausea and vomiting observed.

## Discussion

This is a detailed volunteer study on the blockade range of the ultrasound-guided ESP block. The results of this study suggest that a 20 ml injection of 0.5% ropivacaine in the erector spinae plane administered to the left side of the T5 transverse process results in a significant block of the ipsilateral dorsal cutaneous sensory block. The cold sensory range of declination was from the T1 down to L4 spinous process, with a concentration at T4-T11, and a lateral effect extending between the posterior median and the posterior axillary line. The anterior chest wall, lateral chest wall and abdominal wall were not affected.

The ESP block we applied, was injected the local anesthesia towards the TP, between transverse process and muscle layers. Actually, there are many clinical modifications to ESP block. Cosarcan SK et al. [13] advanced the needle over the intertransverse ligament, added the injection of local anesthetics above the superior costotransverse ligament (SCTL) to increase the spread into the paravertebral space. Tulgar S et al. [14] demonstrated different injection points of ESP block, costotransverse block (CTB),and mid-transverse process (MTP) block. It should be emphasized that our method of ESP block does not penetrate the intertransverse ligament. There are many problems with the modified ESP blocks mentioned in the article, such as their nomenclature problem, the problem of ligament recognition under ultrasound [14], which is contrary to the simplicity and safety of the traditional ESP block. Therefore, we insist on adopting traditional ESP block towards TP, while the modified ESP blocks are still controversial and out of our research scope.

Oksuz et al. [10] reported that bilateral ESP block after breast reduction is superior to local anesthesia in analgesic drug consumption and pain score. ESP blocks can be used as a suitable, effective and safe method of analgesia after mastectomy. However, Ueshima et al. [15] reported that an ESP block cannot effectively achieve the full analgesia of T2-T6 anterior branch, and cannot be assumed to provide complete analgesia for breast cancer surgery. Drennen et al. [16] questioned the pain scores of Oksuz et al. [10] in that they were higher when compared to previous reports, and questioned the efficacy and use of ESP block in breast reduction surgery without better evidence. Therefore, whether ESP block can produce reliable postoperative analgesia in breast surgery remains uncertain.

A single-center study [3] concluded that the ESP block and thoracic PVB have similar analgesic effects in patients undergoing open thoracic surgery. The advantage of ESP blocks is that they have a low incidence of adverse reactions. Taketa et al. [17] suggested that ESP blocks have the properties of a strong lateral cutaneous branch block that are similar to pectoral nerves (PECS) block, but not to PVB or ICNB. Whether ESP blocks can be effectively used in thoracoscopic surgery is still in dispute. Some reports have indicated [6, 11, 18] ESP blocks can be used for abdominal surgery.

Currently most clinical studies support the use of ESP blocks for postoperative analgesia in surgeries involving the posterior thorax, chest, and abdomen. The literature [1, 19, 20] indicates that the specific mechanism of the ESP block involves local anesthetic diffusion in a cephalad-to-caudal direction in the erector spinae plane. It may enter the paravertebral space through the connective tissue complex attached to the transverse process, and then on through the intervertebral foramen. Therein, the ventral and dorsal branches of the spinal nerve are blocked. Gaweda et al. [9] conducted a RCT study that showed the addition of PECS block to ESP block improved postoperative pain control and increased patient satisfaction. If the mechanism of ESP block is diffusion to the paravertebral space, the results for both groups should be the same. Nuclear magnetic resonance studies by Adhikary et al. [21] and Schwartzmann et al. [20] suggested that contrast agents can enter the paravertebral space, and even the epidural space. Further cadaveric studies [22, 23] also suggested that dyes can enter the paravertebral area. However, other studies have raised concerns and objections. Otero PE et al. [24] according to inject dye in the erector spinae plane in a porcine living model, found there is no evidence of anterior spread of dye involving thoracic paravertebral or epidural spaces. The cadaver study by Ivanusic et al. [25] also demonstrated that there was no spread of dye anteriorly to the paravertebral space to involve the origins of the ventral and dorsal branches of the thoracic spinal nerves. Therefore, the theory of paravertebral diffusion and intercostal nerve block is not supported. They speculated that the lateral thoracic wall block may be related to the lateral diffusion of local anesthetic drugs affecting the lateral cutaneous branch of the intercostal nerve [25]. Taketa et al. [17] also confirmed the loss of cold sensation and the disappearance of acupuncture pain when measured at the anterior axillary line and the midclavicular line during an ESP block. Additionally, CT reconstruction by Forero et al. [1] and the use of nuclear magnetic resonance by Schwartzmann et al. [20] indicated that contrast media has limited diffusion to the lateral side. Adhikary et al. [21] made anatomical observations suggesting that in ESP blocks, the dye diffusion was within 10 cm from the posterior median line, thereby not supporting the theory of the lateral spread of local anesthetic drugs. There still remains contradictory evidence regarding paravertebral diffusion in ESP blocks between the different cadaveric studies and medical imaging studies.

Our volunteer trials suggested that ultrasound-guided ESP block only affects the dorsal branch of the ipsilateral spinal nerve. There was no evidence of the block affecting the paravertebral space, intercostal nerve, or lateral branch of the intercostal nerve. Nevertheless, we cannot exclude other anatomical mechanisms, independent of diffusion to the paravertebral space, which could result in the analgesic efficacy of the ESP block observed in clinical practice.

The thoracolumbar fascia, which is a complex structure of several layers that forms a girdle with a sustaining and stabilizing function, seems to contain a high density of nerves and sympathetic fibers [26]. The diffusion of local anesthetic drugs in the thoracolumbar fascia helps to modulate the somatic and visceral pain. Recently, Otero PE et al. [24] found that the thoracic lymph nodes were stained when exploring the mechanism of ESP block in a porcine model. They considered it might be that the local anesthesia entered the lymphatic reflux and desensitized the sensory nerve fibers connected to the lymphatic finger, contributing to clinical analgesic effects. Regardless of the mechanism, further clinical research is needed to apply ESP block to thoracoabdominal surgery.

However, the ESP block does produce a definite block in the dorsal branch of the spinal nerve, and can be used in pediatric oncological posterior thoracic [27] and posterior cervical surgery [28]. The future research directions of ESP block use should study the effect of high-thoracic ESP block in scapular surgery and cervical dorsal surgery, and the effect of middle and lower thoracic ESP blocks in thoracic vertebral surgery.

There are several limitations in our study: 1) this is a volunteer study and the sample size is small. There was not a large enough sample size to study drug concentration, capacity, and the effect of the patient’s position on drug diffusion, 2) we only used cold stimulation to measure the cold sensation range. The pain sensitivity and tactile sensation were not measured, and the disappearance and decrease of the cold sensation were biased due to the subjective feelings of the volunteers.3) we didn’t adopt modified ESP blocks, modified ESP blocks may lead to different results.

## Conclusion

Ultrasound-guided ESP blocks using 20 mL of 0.5% ropivacaine can provide a widespread cutaneous sensory blockade of posterior chest wall, while sparing the anterior and lateral chest walls, and the abdominal wall. The range of blockade suggested only that the dorsal branch of spinal nerve was blocked.

## Data Availability

All data generated or analysed during this study are included in this article.

## Abbreviations

ESP: Erector spinae plane
CSLA: Cutaneous sensory loss area
CSDA: Cutaneous sensory declination area
PVB: Paravertebral block
RCT: Randomized controlled trial
LC: Laparoscopic cholecystectomy
ICNB: Intercostal nerve block
ASA: American Society of Anesthesiologists
BMI: Body mass index
TP: Transverse process
SCTL: Superior costotransverse ligament
CTB: Costotransverse block
MTP: Mid-transverse process
PECS: Pectoral nerves
